# The Prevalence and Determinant of PTSD Symptoms among Home-Quarantined Chinese University Students during the COVID-19 Pandemic

**DOI:** 10.3390/healthcare9101383

**Published:** 2021-10-16

**Authors:** Yueyang Zhang, Jingjing Zhao, Juzhe Xi, Bingbing Fan, Qiong Wang, Zhiying Yao, Tianhui Huang, Han Bai

**Affiliations:** 1 Department of Biostatistics, School of Public Health, Cheeloo College of Medicine, Shandong University, Jinan 250012, China; 201800220076@mail.sdu.edu.cn (Y.Z.); bingbingfan@mail.sdu.edu.cn (B.F.); 2 School of Public Health, Cheeloo College of Medicine, Shandong University, Jinan 250012, China; 3 Shanghai Key Laboratory of Mental Health and Psychological Crisis Intervention, Affiliated Mental Health Center (ECNU), School of Psychology and Cognitive Science, East China Normal University, Shanghai 200062, China; jzxi@psy.ecnu.edu.cn; 4 Centre for Health Management and Policy Research, School of Public Health, Cheeloo College of Medicine, Shandong University, Jinan 250012, China; 202020815@mail.sdu.edu.cn; 5 Department of Epidemiology, School of Public Health, Cheeloo College of Medicine, Shandong University, Jinan 250012, China; 201915777@mail.sdu.edu.cn; 6 Paul H. Nitze School of Advanced International Studies, Johns Hopkins University, Baltimore, MD 21218, USA; thuang50@jh.edu; 7 Department of Health Policy, School of Medicine, Stanford University, Stanford, CA 94305, USA; hanbai@stanford.edu

**Keywords:** PTSD, determinant, prevalence, university students, COVID-19

## Abstract

Background: When COVID-19 emerged in China in late 2019, most Chinese university students were home-quarantined to prevent the spread of the virus, considering the great impact of the lockdown on young people habits and their psychological well-being. This study explored the prevalence of post-traumatic stress disorder (PTSD) and its associated factors among Chinese university students who are isolated at home during the COVID-19 pandemic. Method: 4520 participants from five universities in China were surveyed by online questionnaire and the PTSD Checklist—Civilian Version (PCL-C) was adopted as a screening instrument. Results: Exposure to virus was significantly related to PTSD outcomes. The most important predictors for PTSD outcomes were parents’ relationship and the way parents educated, and university-provided psychological counseling was a protective factor against developing PTSD. Conclusions: The COVID-19 pandemic had adverse psychological consequences on Chinese university students who were isolated at home due to the relatively high prevalence rate of PTSD which was reported. Adverse parental relationships and the extreme way parents educate their children could be the major risk factors for PTSD outcomes. Psychological interventions need to be made available to home-quarantined university students, and those in the worst-hit and exposed areas to virus should be given priority focus.

## 1. Introduction

In December 2019, the Coronavirus Disease 2019 (COVID-19) epidemic broke out in Wuhan, China [1]. Being highly transmissible, COVID-19 has spread widely around the world by multiple routes [2]. In addition, some relevant researchers believe that the COVID-19 pandemic is expected to last for a long time [3], posing a serious threat to public health and safety. As of 9 August 2020, the total number of confirmed cases of the coronavirus has exceeded 1.78 million globally with the cumulative number of deaths exceeding 728,000 [4].

It is well known that stressful events, such as natural disasters and man-made traumas, can affect the mental health of the public and may even lead to the development of mental disorders [5,6,7]. Post-traumatic stress disorder (PTSD) is one such disorder that can develop from an individual’s contact with traumatic events. PTSD’s characteristic symptoms are the re-experience of the traumatic event, avoidance of activities and situations that are easy to associate with said trauma, repeated numbness, increased alertness, and memory and cognition problems [8].

PTSD is a kind of chronic damage disease, which carries certain harm to an individual’s psychology and physiology and can cause harm to their family and society. However, there are few cross-sectional studies on PTSD under the influence of the pandemic [9]. Many studies during the COVID-19 pandemic have shown that medical staff, COVID-19 patients, and other populations have different levels of psychological problems, demonstrated as worry, fear, PTSD, depression, etc., but there are few studies on the psychological impact of COVID-19 on the average Chinese university student who is still in the mature period of psychological development. Since the early days of the pandemic, public health experts have noted that the prevalence of PTSD in the general population may increase [10]. The demand for psychological intervention will grow, and research on related psychological effects will be urgently required [11]. In view of the limited information obtained from previous studies, more research is needed to understand the impact of the COVID-19 pandemic on people’s psychology.

The population of Chinese university students is very large. There are nearly 2300 universities in China and nearly 30 million university students [12]. As COVID-19 continues to spread around the world, accompanied with more infections, the epidemic has become normalized. It presents as an uncontrollable and continuous source of stress compared with other common stressors in life. Therefore, given the current situation, studies on the prevalence and risk factors for potential mental health problems can assist schools and families in coping with the possible negative psychological consequences of pandemics on the general university student population.

Long-term isolation at home allows university students to spend more time with their parents. Previous studies have shown that a toxic family situation will raise the prevalence of psychological problems among family members [13,14,15]. Adolescents with good family relationships exhibit closeness and positive reciprocity among family members. Positive relationships within the family can provide protection, resilience, effective communication, and parenting, and improve the mental health of young people [16,17]. Therefore, a student’s family situation affects their mental health and the prevalence of mental illness during the pandemic. One’s family situation is very important as a predictor of one’s susceptibility to PTSD [18]. However, no one has studied the relationship between family factors and student mental illnesses through the lens of the COVID-19 pandemic. Therefore, it is necessary to understand the possible relationship between the family situation in the backdrop of COVID-19 pandemic and potential mental illnesses such as PTSD of university students for successful family intervention.

China has redoubled its efforts to prevent the coronavirus from re-entering and spreading within the country after the outbreak. Some studies during the SARS epidemic have revealed that people’s psychological state is related to virus exposure [19,20]. Higher levels of disease exposure and fear are significantly associated with worse mental health outcomes and PTSD [21]. Therefore, it is necessary to understand the relationship between virus exposure and mental illness during the COVID-19 pandemic and to understand the psychological impact of major outbreaks on people’s mentality.

During the pandemic, some universities in China have implemented psychological intervention measures for students, which yielded good results. However, few studies have been conducted to quantify the impact of implementing intervention measures on university students’ psychology during the new COVID-19 pandemic. Therefore, this study aims to quantify the relationship between the intervention measures imposed by universities and the PTSD of university students during the pandemic so as to screen practical measures. This study is of practical significance and lays a theoretical basis for practice.

## 2. Materials and Methods

### 2.1. Participants and Procedure

This cross-sectional study for this project on undergraduate students using multistage sampling and stratified sampling was conducted in five universities, including Shandong University, Liaocheng University, Yantai University, Jining College, Weifang University. A total of 4520 undergraduates from freshmen to senior of different subjects (arts, science, medicine, and agriculture, etc.) in 30 colleges at the five universities were included in the survey. One class from each grade of each major was randomly selected, respectively.

Because of the limited resources and social distancing policy adopted, the survey was completed through the Chinese Surveystar website from 8 April 2020 to 24 April 2020, approximately three months after China declared a state of emergency due to COVID-19. We contacted the subjects by WeChat, which is a commonly used social application in China. We also contacted the full-time tutors/parents of our sampled colleges and classes to send out the link of electronic questionnaire in their WeChat class group. Full-time tutors/parents were trained online to help the students during the survey. We only recruited minor university students with the consent of the relevant minor university students’ parents. Statements of the purpose of the research and assurance of the confidentiality and privacy of participating individuals were placed on the first page of the survey questionnaire. Participants could only complete the questionnaire after reading this statement by clicking “AGREE” to confirm their consent. All participants were told that they had the right to stop the survey at any time. Each student who completed the survey also had the chance to receive random WeChat Lucky Money of CNY 2–5. The survey was anonymous, but all participants were asked to give the last four digits of their phone numbers as the survey was to be used again in the future.

### 2.2. Measures

#### 2.2.1. Sociodemographic Variables

Demographic data, including gender, age, university year, major, ethnicity, student leadership status, membership status with the Chinese Communist Party (CCP), and place of birth were collected via survey from each participant.

#### 2.2.2. PTSD Symptoms

The PTSD Checklist—Civilian Version (PCL-C) is a self-report rating scale for PTSD [22], comprising 17 items that assess the full domain of DSM-IV PTSD symptoms. The questionnaire covers three symptom clusters of PTSD: re-experiencing symptoms (five items), seven numbing/avoidance symptoms (seven items), and hyperarousal symptoms (five items), DSM-IV criteria B, C, and D, respectively [23]. It asks respondents to rate past-month symptoms of PTSD based on referent to “a past stressful experiences” on a 1–5 Likert scale, with 1 = not at all, 2 = a little bit, 3 = moderately, 4 = quite a bit, and 5 = extremely. Responses on the PCL-C can be used in two ways to arrive at a diagnosis of PTSD [22].

A total symptom severity score can be obtained by summing the scores from each of the 17 items. The recommended cutoff score of 44 for the scale suggests a PTSD diagnosis. All responses generate a total score range from 17–85 and higher scores indicate higher PTSD levels [24,25,26]. Scoring can also be completed using the symptom cluster method; if the participant endorses at least one B item (re-experiencing symptoms, questions 1–5), three C items (numbing/avoidance symptoms, questions 6–12), and at least two D items (hyperarousal symptoms, question 13–17) with a rating of 3 or above (i.e., a score of 3 or more on a 5-point scale), PTSD symptoms are considered to be present. Although either scoring method can help determine a diagnosis, this study used the symptom cluster method of evaluation. This scale has demonstrated good internal and test–retest reliability in a Chinese sample [27]. In the current study, Cronbach’s α was 0.965.

#### 2.2.3. Exposure

A COVID-19 exposure scale was adapted from a modified version of the disaster exposure severity scale based on DSM-IV criteria for PTSD. The scale included thirteen objective items coded as yes or no questions: (1) Have you traveled outside this area; (2) whether there are medical workers around (family members, neighbors, relatives); (3) whether any family members support the Hubei Medical Team; (4) whether any family members participate in local pandemic prevention work; (5) whether you participate in local pandemic prevention work; (6) whether there are confirmed cases in your villages/community; (7) whether there are confirmed cases in your township/town/street; (8) whether there are confirmed cases in your county/district; (9) whether there are confirmed cases in your city; (10) whether there are imported cases from abroad in your village/community; (11) whether there are imported cases from abroad in your township/town/street; (12) whether there are imported cases from abroad in your county/district; (13) whether there are imported cases from abroad in your city. The total score based on the objective and subjective experience was calculated by adding up the yes responses.

#### 2.2.4. Family Factors

Family factors were classified into three parts, which were family background (the first part), basic characteristics of parents (the second part), and relationships between family members (the third part). The three parts above proved to be the main family factors affecting the psychological state of university students based on existing research [28]. Family type, financial situation, and parenting style belong to family background. Parents’ academic credentials belong to the second part, and the relationship between parents belongs to the third.

#### 2.2.5. University’s Response to COVID-19

In response to the pandemic, universities have also taken relevant measures, and some of the measures were selected in study as the independent variables, including (1) did the university require students to report daily information; (2) did the university popularize scientific information on COVID-19; (3) did the university adopt virtual learning; (4) did the university provide psychological counseling?

### 2.3. Statistical Analysis

Differences in frequencies and proportions were tested using the chi-square test. Binary logistic regression was utilized to explore the association between the variables and PTSD total score. Four multivariable logistic regression models were employed to examine the associations between PTSD and various sociodemographic variables, exposure, family factors, the university’s response to COVID-19 among undergraduate students, with a *p*-value of less than 0.05 being considered statistically significant. Data analyses were performed using the SPSS software package (SPSS 22.0, SPSS Inc., Chicago, IL, USA).

### 2.4. Quality Control

Each Internet Protocol (IP) address could be used only once to answer the questions. After all questionnaires were collected, invalid questionnaires were eliminated according to the following criteria: (1) all the questions were completed in less than 120 s; (2) the demographic information was incomplete or irrelevant to the survey, and (3) logical inconsistency was found in answers.

### 2.5. Ethics Approval

All subjects gave their informed consent for inclusion before they participated in the study. The study was designed in accordance with the tenets of the Declaration of Helsinki, 1996, and was approved by the ethical committee of Shandong University before data collection (Task No. LL20200201).

## 3. Results

### 3.1. The Result of Descriptive Analysis of Independent Variables

Demographic characteristics of the sample and descriptive data on all predictors and outcomes are shown in Table 1. In total, 3961 participants were included in our analysis due to 559 subjects not following the data quality control criteria, including 2454 (62.0%) females and 1507 (38.0%) males. Concerning age, there were three age groups with 1098 students between the ages of 16 and 19, 2795 between 20 and 23, and 68 aged 24–27, respectively. Of the total sample, *n* = 1012 (25.5%) were in the first year of university, *n* = 1036 (26.2%) were in the second year, *n* = 943 (23.8%) were in the third year, and *n* = 970 (24.5%) were in the fourth year. The majority of respondents were ethnically Han (96.4%), non-student leaders (74.2%), non-Party members (94.4%), non-natives of Hubei (98.9%). Additionally, 19.7% of the students majored in engineering, 28.1% in science, 4.3% in agriculture, 30.2% in liberal art, 3.6% in medicine, and 14.2% in arts.

### 3.2. The Result of Exposure to COVID-19

Reported exposure to COVID-19 was as follows: 6.2% reported that they had traveled outside their cities; 18.2% reported that there were medical workers around (family members, neighbors, relatives) them; 15% reported that their family members joined medical teams supporting Hubei; 19.5% reported that their family members participated in local epidemic prevention work; 6.5% reported that he/she participated in local epidemic prevention work; 8% reported confirmed cases in their communities; 23.3% reported confirmed cases in the streets of their towns and townships; 66.3% reported confirmed cases in the their counties; 89.5% reported confirmed cases in their cities; 4.5% reported imported cases in their communities; 11.5% reported imported cases in their townships/towns/streets; 29.6% reported imported cases in their counties/districts; 55.2% reported imported cases in their cities.

### 3.3. The Result of Family Factors

The family factors were as follows. In response to the question “What is your family type?” participants indicated whether they were living in a nuclear family household (*n* = 3664, 92.5%), an extended family household (*n* = 211, 5.3%), a single-parent household (*n* = 70, 1.8%), or a step-family household (*n* = 16, 0.4%). When asked about the family’s financial situation, survey respondents chose from extremely poor (*n* = 225, 2.7%), poor (*n* = 1447, 36.5%), average (*n* = 2239, 56.5%), or rich (*n* = 50, 1.3%). Results about parenting styles showed authoritarian parenting (*n* = 338, 8.5%), neglectful parenting (*n* = 207, 5.2%), democratic parenting (*n* = 3352, 84.6%), or permissive parenting (*n* = 64, 1.6%). Fathers’ education levels consisted of high school completion and below (*n* = 3198, 80.7%), Bachelor’s degree (*n* = 707, 17.8%), Master’s degree and above (*n* = 56, 1.4%). While in mothers, a result of high school completion and below (*n* = 3378, 85.3%), Bachelor’s degree (*n* = 556, 14.0%), Master’s degree and above (*n* = 27, 0.7%) was observed. Relationship between parents: alienated (*n* = 71, 1.8%), plain (*n* = 794, 20.0%), more harmonious (*n* = 2022, 51%), very affectionate (*n* = 1074, 27.1%).

### 3.4. The Result of University Responses to COVID-19

The universities’ response to COVID-19 was as follows: 92% reported that their universities required students to upload specific information every day; 91.6% reported that their universities popularized scientific information on COVID-19; 84.3% reported that their universities adopted online learning; 67.3% reported that their universities provided psychological counseling.

### 3.5. PTSD Symptoms

We used the recommended PCL-C symptom cluster method, and the prevalence rate of PTSD in our sample was 22% (*n* = 832). As shown in Table 2, the positive rates of B, C, and D, namely the symptoms of re-experiencing symptoms, avoidance symptoms, and excessive arousal symptoms were 43.2, 28.6, and 32.9%. The positive rates of 17 items ranged from 20.1 to 37.4%, with relatively high rates observed in recurring psychological epilepsy (35.81%), recurring traumatic events (28.94%), and trying to avoid thinking or talking about infections (26.69%) experienced most often.

### 3.6. The Relationship between Demographic Characteristics, Family Factors, Exposure Factors, and PTSD

The χ2 test was used to analyze the relationship between demographic characteristics, exposure, family factors, university’s response to COVID-19, and PTSD. It found that the prevalence of PTSD among university students of different genders was statistically different, and the prevalence of PTSD of men was higher than that of women (χ2 = 33.301, *p* < 0.001). The prevalence of PTSD of university students in different majors was statistically different; those majoring in art had the highest positive rate of PTSD, and those majoring in engineering had the lowest (χ2 = 11.305, *p* = 0.046); the prevalence of PTSD in university students who are not student leaders was higher than university students who are student leaders (χ2 = 7.52, *p* = 0.006); university students with confirmed cases in the residential village community exhibited higher prevalence of PTSD than those without (χ2 = 13.645, *p* < 0.001); students with imported cases in the village/community/community displayed higher prevalence of PTSD than those without (χ2 = 23.782, *p* < 0.001); the prevalence of PTSD in university students with imported cases in the township/town/street of residence was higher compared with students without imported cases (χ2 = 7.207, *p* = 0.007); the prevalence of PTSD among university students from different family types was statistically different. The prevalence of PTSD peaked among university students from step-families, and the prevalence of PTSD among students from nuclear families reached the lowest (χ2 = 8.772, *p* = 0.032); the prevalence of PTSD among university students with different family financial situations was statistically different. University students from extremely poor families had the highest prevalence of PTSD, in contrast to the lowest level among students from wealthy families (χ2 = 25.827, *p* < 0.001); significant difference was detected in the prevalence of PTSD among university students with different parenting styles. University students with permissive parenting had the highest positive rate of PTSD, and those with democratic parenting the lowest (χ2 = 50.717, *p* < 0.001); there were statistical differences in the prevalence of PTSD among college students with different fathers’ education levels. Students whose fathers completed high school or failed to do so reported the highest prevalence of PTSD, and students whose fathers received a Master’s degree or above the lowest (χ2 = 6.238, *p*= 0.044); the prevalence of PTSD of university students with different parental relationships was statistically different. The prevalence of PTSD of university students whose parental relationship was alienated and excluded reached the highest level, and that of students whose parental relationship was very affectionate was the lowest (Χ2 = 78.727, *p* < 0.001); students whose schools required daily uploading of specific information (χ2 = 7.509, *p* = 0.006), popularized scientific information on the virus (χ2 = 17.055, *p* < 0.001), embraced virtual learning (χ2 = 5.811, *p* = 0.016) and psychological counseling (χ2 = 23.564, *p* < 0.001) showed lower prevalence than those whose schools did not. See Table 3 for details.

### 3.7. Comparative Analysis and Univariate Logistic Regression of the PTSD Symptom Stratified by Demographic Characteristics, Exposure, Family Factors, and University’s Response to COVID-19

To better explore the relationship between the occurrence of PTSD and various influencing factors, we analyzed the results by exploiting four models (Table 4). Only demographic characteristics were introduced in Model 1. Model 2 included exposure factors, in both domestic and imported cases. Family factors were added in Model 3. Measure factors were incorporated in Model 4. The research results showed that there was significant correlation between demographic characteristics, exposure factors of imported cases, family factors, measurement factors, and regression of PTSD occurrence. Specifically, being female (*p* < 0.001, OR = 0.621), having a democratic deliberative upbringing (*p* = 0.017, OR = 0.73), having a very affectionate the relationship with one’s parents (*p* < 0.001, OR = 0.309), and psychological counseling provided by the school (*p* = 0.004, OR = 0.766) were the protective factors against developing PTSD. Being an art student (*p* < 0.001, OR = 1.76) and seeing imported cases of the coronavirus in the residential village/community/community (*p* = 0.009, OR = 1.725) were the risk factors for developing PTSD.

## 4. Discussion

This study evaluated the prevalence of PTSD in home quarantined university students about 4 months after the outbreak of COVID-19 in China and identified relevant risk factors.

The prevalence of PTSD among university students surveyed was 22%, which was higher than that in other studies in China. In a study conducted two weeks after the declared start of the COVID-19 pandemic, 14.4% of the sample population of 584 young people were reported to have PTSD [29]. More early data from the COVID-19 pandemic showed that PTSD and trauma symptoms have been prevalent in the general population since the COVID-19 outbreak [30,31,32]. The increase in rates may result from continued stress as the COVID-19 pandemic normalizes. A meta-analysis concluded that the average incidence of PTSD in the general population during the COVID-19 pandemic was 8.6% [33]. The prevalence of PTSD found in this study was higher than these average estimates. This was likely due to the age of the subject pool. Because the subject pool consisted of university students who were still in early adulthood, not yet mature, and in stages of cognitive development, participants were at higher risk of developing mental disorders and mental illnesses, especially when encountering public emergencies. After a crisis, their mental state was expected to be more vulnerable than that of adult age groups.

An American cross-sectional study of 898 young people (18–30 years) one month after the U.S. declared a state of emergency due to COVID-19 found that the prevalence of PTSD in American young adults was 31.8% [9], much higher than the 22% prevalence rate found in our study. These differences may be attributed to different exposure conditions. The United States did not take sufficient effective isolation measures in the early stage of the COVID-19 pandemic [34]. In addition, the current COVID-19 pandemic has entered a period of normalization [35]. Due to the high prevalence of PTSD among university students and the presence of continuous stressors, it was recommended that university mental health professionals, psychosocial institutions, and the government pay more attention to the PTSD status of university students so that they can provide relevant guidance where needed.

This study showed that males had a higher risk of developing PTSD than females. This was inconsistent with the results of other studies [36,37]. A meta-analysis on the prevalence of PTSD among Shidu parents who lost their children found that Shidu mothers had higher PTSD scores than Shidu fathers (SMD [95%CI]: 0.41 [0.20, 0.62], *p* < 0.001) [38]. A study about the prevalence of PTSD among adolescents exposed to natural disasters indicated that the prevalence of PTSD in women was higher than that in men [39], and a survey of the psychological impact of the COVID-19 on the general population stated that the prevalence of PTSD in women was higher than that of men [40]. The differences may be due to differences in the target population, age, stressful events revealed, and stressors, or the different ways of men and women to cope with trauma. Previous research showed females had a higher prevalence of PTSD 1 month after COVID-19 [41]. The same result was also confirmed 2 months after COVID-19 [42]. Chinese females might find it easier to express their symptoms indicating PTSD in the short term after the earthquake; however, this expression is a particularly effective way to vent and reduce PTSD, and so short-term studies would find female survivors reported higher incidences of PTSD, but males reported higher incidences at later time points [43].

In the four models, art students were shown to be more likely to suffer from PTSD than engineering students. It may be that art majors’ students have strong perceptual thinking that may lead them to be more likely to develop PTSD. Given the creative nature of art students, many are often socially active, extroverted, empathetic, and sensitive to their surroundings. Their sensitive and impulsive nature may result in their emotional and mental states taking on heavier tolls in response to changes in society and the surrounding environment. In times such as the COVID-19 pandemic where sudden societal and environmental change is occurring, art students may be affected more than other students, making them more susceptible to developing PTSD.

By mid-April 2020, the Chinese domestic epidemic situation had gradually improved, but COVID-19 was still a global concern with the condition outside China becoming more and more severe. The number of cases imported from abroad into China has gradually increased. On 17 April 2020, there were 31 new cases in China, and 85,383 new cases in countries outside of China. On 22 April, there were a total of 2,490,701 confirmed cases in countries outside of China. On 26 April, there were 1192 confirmed cases in China, and 1634 imported cases from abroad. On 30 April, there were 914 confirmed cases in China and 1664 imported cases outside China [44]. Studies have shown that the long-term presence of the virus in an organism can cause myriad types of mutations [45]. By 21 April 2020, a study revealed that COVID-19 has mutated into 30 different strains, which was found distributed in various epidemic countries [46]. The continued importation of foreign cases and the rapid mutation of the virus may have a negative psychological impact on the students. This seemed to explain why a higher prevalence of PTSD was associated with imported cases in the residential village/community instead of domestic confirmed cases in the residential village/community. Some previous studies believed that trauma exposure was the primary factor affecting people’s post-traumatic physical and mental response [47]. Other studies suggested that disaster exposure was significantly related to the symptoms of post-traumatic stress disorder in adolescents [48], and the closer to the center of the crisis event, the higher people’s negative emotions were towards the event [49]. For example, in the results of this study, the presence or absence of imported cases in the township/town/street of residence was irrelated to PTSD, a possible explanation of which was that people live far away from the confirmed patient. It can be shown, under the given conditions, being in close proximity to a confirmed patient was a risk factor for PTSD, which was consistent with the results of previous studies [50]. Perhaps because residents closer to the confirmed patients were more susceptible to the virus, individuals feel more threatened by the situation. Thus, their subjective threat of trauma was stronger [51,52]. In addition, neighbors in the community or family members at risk of contacting suspected or confirmed patients coupled with fear that family members or themselves will be infected leads to more negative emotions under such circumstances. Moreover, there were asymptomatic infections with an incubation period of up to 14 days, which made epidemic prevention and control increasingly difficult and created mounting panic in the community.

Due to the tough isolation and lockdown measures adopted by China, universities delayed opening but provided online courses for students at home. Related studies have suggested that the mental health status of people in isolation was correlated to the number of cohabitants [14]. There were also studies that suggest it takes less than one year for the symptoms of PTSD in adolescents to affect conflict communication between parents and children. Additionally, family dynamics can predict PTSD symptoms in adolescents 20 months in the future [53]. People with poor mental health also experienced more family conflicts [14]. Therefore, family conditions may affect the mental health of university students and the incidence of PTSD during the pandemic. Of the research sample, 92.5% were nuclear families. Excluding cohabitation with grandparents and other relatives, the basic members of a household were parents and children, which increased the importance of research on family factors.

Previous studies have also shown that one’s family education method was also an influencing factor that caused problems in the mental health of college students. This was caused by long-term psychological penetration. A parent’s every move may affect the behavior and thoughts of students in a subtle way. Among them, the manipulative education model had a greater impact on the individual’s psychology, causing a great psychological burden on the child, and easily causing them to feel uneasy and anxious. This may produce more psychological problems as the child grows up [54,55]. This study agreed with that expectation. During the COVID-19 pandemic, students whose parents’ education methods were strict and controlling experienced greater psychological problems and a higher incidence of PTSD. Because authoritarian parents want their children to act in accordance with their wishes, they inadvertently suppress their children’s personalities to a large extent. As a result, their kids may exhibit aggressive and withdrawal behaviors. This kind of education method poses hidden risks to children’s mental health, increasing the prevalence of PTSD in the sample population during the pandemic. Studies have also found that the mental health of students with harmonious parental relationships was significantly better than that of students with normal and discordant parental relationships [56]. A higher incidence of PTSD was found in university students who had strained relationships with family. Research has also found that the total average score and interpersonal sensitivity factors of the Chinese students with a disharmonic relationship with parents were very significantly higher than the students with a harmonious parental relationship. Parental discord may directly lead to the decline of children’s mental health with the degree of harm even exceeding that associated with parental divorce [56]. It may be that the cold war or quarrel caused by the rejection of the parental relationship creates an environment that lacks warmth for children, which leads to a decrease in the children’s sense of security and engenders negative emotions. This may affect a child’s mental health in the long term. In situations such as during a pandemic, multiple internal and external stressors and other related pressures will make the situation worse. Of course, there were also studies suggesting that sudden public health emergencies can cause parents to have negative psychological emotions. These reactions may negatively affect their children and cause the children to reciprocate emotionally [39].

The results of this study showed that the prevalence of PTSD was low among university students whose university provided psychological counseling measures during the COVID-19 pandemic. Having access to counseling resources was a protective factor against developing PTSD symptoms. Correctly helping university students implement relevant emotional strategies and identifying and resolving psychological problems can prevent or alleviate mental health problems related to fatal pandemic events. It can reduce the degree of panic and severity of mental illness in students brought about by public health events [57]. Psychological education and social mentality guidance in universities were of great value in response to the pandemic [58].

This research aimed to guide us to pay attention to the psychological situation of males and students majoring in art. It was important for parents to pay attention to the psychological demands of their children, create a good family environment, and adopt a democratic and deliberative approach to education. At the same time, it was necessary to provide parents with relevant education and psychological counseling during the pandemic to raise awareness of their children’s mental states and help reduce the likelihood of their children developing mental illnesses. These proposed changes to existing interventions may enhance the effectiveness of treatments related to stress responses in parents and adolescents after disasters. We should focus on the students who have been severely exposed to the epidemic, raise the awareness of the epidemic situation, and strengthen psychological counseling in universities.

## 5. Limitation

We should understand that this study has certain limitations. First, some other variables, such as coping style, the length of isolation time, and other potential factors may affect the results, which were not measured in this study. Second, this online study is based on self-assessed data, which are highly subjective and may result in recall bias. Third, the study results concluded from college students may not be applicable to other populations such as adults or the elderly. Fourth, the use of a cross-sectional design cannot provide strong evidence of causality.

## 6. Conclusions and Implications

This study can help identify the occurrence of PTSD among university students so that appropriate psychological interventions can be taken in a targeted manner. We found that Chinese university students who are isolated at home had adverse psychological consequences and higher incidence of PTSD during the COVID-19 pandemic. Adverse parental relationships and the extreme way parents educate their children could be major risk factors for PTSD outcomes. Psychological interventions need to be made available to home-quarantined university students, and those in the worst-hit and exposed areas to the virus should be given priority focus. We should list the above-mentioned people as important objects of psychological counseling. In addition, as the epidemic becomes normalized, more research should be carried out to determine the PTSD conditions of university students in different time periods and different countries to provide data in a wider range. The current research results may provide basic information for the mental health programs for university adults and other groups.

## Figures and Tables

**Table 1:** Descriptive analysis of independent variables (*n* = 3961).

Variable	Classification	Total (*n* = 3961)
Gender	Male	1507 (38.0)
	Female	2454 (62.0)
Age	16–19	1098 (27.7)
	20–23	2795 (70.6)
	24–27	68 (1.7)
University Year	1st year	1012 (25.5)
	2nd year	1036 (26.2)
	3rd year	943 (23.8)
	4th year	970 (24.5)
Major	Engineering	780 (19.7)
	Science	1114 (28.1)
	Agriculture	169 (4.3)
	Liberal art	1195 (30.2)
	Medicine	141 (3.6)
	Art	562 (14.2)
Ethnicity	Han	3817 (96.4)
	Minority	144 (3.6)
Student leader	Yes	1021 (25.8)
	No	2940 (74.2)
Chinese Communist Party (CCP) member	Yes	220 (5.6)
	No	3741 (94.4)
Born in Hubei	Yes	44 (1.1)
	No	3917 (98.9)
History of outings	Yes	247 (6.2)
	No	3714 (93.8)
Surrounded (family members, neighbors, relatives) by medical workers	Yes	719 (18.2)
	No	3242 (81.8)
Some family members participated in supporting the Hubei Medical Team	Yes	15 (1.4)
	No	3946 (99.6)
Some family members are involved in local pandemic prevention	Yes	771 (19.5)
	No	3190 (80.5)
I participate in local pandemic prevention work	Yes	258 (6.5)
	No	3703 (93.5)
Confirmed cases in residential villages/communities	No	3643 (92.0)
	Yes	318 (8.0)
Confirmed cases in residential township/town/street	No	3037 (76.7)
	Yes	924 (23.3)
Confirmed cases in resident county/district	No	1333 (33.7)
	Yes	2628 (66.3)
Confirmed cases have been reported in cities	No	414 (10.5)
	Yes	3547 (89.5)
Imported cases from abroad in residential village/community	No	3781 (95.5)
	Yes	180 (4.5)
Imported cases from abroad in residential township/town/street	No	3507 (88.5)
	Yes	454 (11.5)
Imported cases from abroad in residential county/district	No	2789 (70.4)
	Yes	1172 (29.6)
Imported cases from abroad in city	No	1773 (44.8)
	Yes	2188 (55.2)
Family type	Nuclear family	3664 (92.5)
	Extended family	211 (5.3)
	Single-parent family	70 (1.8)
	Remarried family	16 (0.4)
Family’s financial situation	Extremely poor	225 (5.7)
	Poor	1447 (36.5)
	Average	2239 (56.5)
	Rich	50 (1.3)
The way parents educate their children	Strict control type	338 (8.5)
	Hands-off type	207 (5.2)
	Democratic deliberative type	3352 (84.6)
	Doting and pampering type	64 (1.6)
Father’s academic credentials	High school degree and below	3198 (80.7)
	Bachelor’s degree	707 (17.8)
	Master’s degree and above	56 (1.4)
Mother’s academic credentials	High school degree and below	3378 (85.3)
	Bachelor’s degree	556 (14.0)
	Master’s degree and above	27 (0.7)
The relationship between parents	Marginalized and alienated	71 (1.8)
	Flat and common	794 (20.0)
	Harmonious	2022 (51)
	Very affectionate	1074 (27.1)
University requires students to report daily information	No	317 (8)
	Yes	3644 (92)
University carried out popular science propaganda	No	333 (8.4)
	Yes	3628 (91.6)
University conducts distance learning	No	620 (15.7)
	Yes	3341 (84.3)
University provides psychological counseling	No	1296 (32.7)
	Yes	2665 (67.3)

**Table 2:** PCL-C quantified form for frequency of different symptoms (N = 3961).

PCL-C Symptoms	No.	%
Re-experiencing symptoms	1712	43.2
1. Having upsetting memories about the trauma	1483	37.4
2. Experiencing bad dreams and nightmares about the event	837	21.1
3. Feeling as if the trauma were happening again	990	25.0
4. Getting depressed when reminded of the event	1154	29.1
5. Reacting physically (e.g., sweating, heart racing, trouble breathing) when reminded of the trauma	797	20.1
Avoidance symptoms	1131	28.6
6. Avoiding trauma-related feelings, thoughts, or conversations	1025	25.9
7. Avoiding places, activities, or people that reminded you of the traumatic event	1027	25.9
8. Trouble recalling important aspects of what happened during the trauma	937	24.6
9. Losing interest in things you used to enjoy	1042	26.3
10. Feeling detached from other people	1053	26.6
11. Feeling emotionally numb	928	23.4
12. Feeling as if your future will be cut short	941	23.8
Arousal symptoms	1304	32.9
13. Difficulty falling or staying asleep	942	23.8
14. Experiencing irritability or outbursts of anger	1062	26.8
15. Trouble focusing on tasks	1374	34.7
16. Feeling constantly alert or always on the lookout for danger	952	24.0
17. Difficulty tolerating and/or easily startled by loud noises	1055	26.6

**Table 3:** The relationship between demographic characteristics, family factors, exposure factors, etc., and PTSD.

Variable	Classification	PTSD Negative	PTSD Positive	χ2	*p*
Gender	Male	1103 (73.2)	404 (26.8)	33.301	<0.001
	Female	1988 (81)	466 (19)		
Age	16~19	868 (79.1)	230 (20.9)	2.185	0.335
	20~23	2174 (77.8)	621 (22.2)		
	24~27	49 (72.1)	19 (27.9)		
University Year	1st year	809 (79.9)	203 (20.1)	7.354	0.061
	2nd year	820 (79.2)	216 (20.8)		
	3rd year	732 (77.6)	211 (22.4)		
	4th year	730 (75.3)	240 (24.7)		
Major	Engineering	638 (81.8)	142 (18.2)	11.305	0.046
	Science	864 (77.6)	250 (22.4)		
	Agriculture	127 (75.1)	42 (24.9)		
	Liberal art	935 (78.2)	260 (21.8)		
	Medicine	106 (75.2)	35 (24.8)		
	Art	421 (74.9)	141 (25.1)		
Ethnicity	Han	2985 (78.2)	832 (21.8)	1.707	0.191
	Minority	106 (73.6)	38 (26.4)		
Student leader	Yes	828 (81.1)	193 (18.9)	7.52	0.006
	No	2263 (77.0)	677 (23.0)		
Chinese Communist Party (CCP) members	Yes	174 (79.1)	46 (20.9)	0.151	0.697
	No	2917 (78.0)	824 (22.0)		
Born in Hubei	Yes	32 (72.7)	12 (27.3)	0.732	0.392
	No	3059 (78.1)	858 (21.9)		
History of outings	Yes	196 (79.4)	51 (20.6)	0.266	0.606
	No	2895 (77.9)	819 (22.1)		
Surrounded (family members, neighbors, relatives) by medical workers	Yes	542 (75.4)	177 (24.6)	3.608	0.057
	No	2549 (78.6)	693 (21.4)		
Some family members participated in supporting the Hubei Medical Team	Yes	10 (66.7)	5 (33.3)	1.136	0.287
	No	3081 (78.1)	865 (21.9)		
Some family members are involved in local pandemic prevention	Yes	592 (76.8)	179 (23.2)	0.876	0.349
	No	2499 (78.3)	691 (21.7)		
I participate in local pandemic prevention work	Yes	192 (74.4)	66 (25.6)	2.107	0.147
	No	2899 (78.3)	804 (21.7)		
Confirmed cases in residential villages/communities	No	2869 (78.8)	774 (21.2)	13.645	<0.001
	Yes	222 (69.8)	96 (30.2)		
Confirmed case in residential township/town/street	No	2389 (78.7)	648 (21.3)	2.989	0.084
	Yes	702 (76.0)	222 (24.0)		
Confirmed case in resident county/district	No	1032 (77.4)	301 (22.6)	0.446	0.504
	Yes	2059 (78.3)	569 (21.7)		
Confirmed cases have been reported in cities	No	313 (75.6)	101 (24.4)	1.595	0.207
	Yes	2778 (78.3)	769 (21.7)		
Imported cases from abroad in residential village/community	No	2977 (78.7)	804 (21.3)	23.782	<0.001
	Yes	114 (68.4)	66 (36.7)		
Imported cases from abroad in residential township/town/street	No	2759 (78.7)	748 (21.3)	7.207	0.007
	Yes	332 (73.1)	122 (26.9)		
Imported cases from abroad in resident county/district	No	2192 (78.6)	597 (21.4)	1.716	0.19
	Yes	899 (76.7)	273 (23.3)		
Imported cases from abroad in city	No	1402 (79.1)	371 (20.9)	2.022	0.155
	Yes	1689 (77.2)	499 (22.8)		
Family type	Nuclear family	2876 (78.5)	788 (21.5)	8.772	0.032
	Extended family	158 (74.9)	53 (25.1)		
	Single-parent Families	47 (67.1)	23 (32.9)		
	Remarried family	10 (62.5)	6 (37.5)		
Family’s financial situation	Extremely poor	163 (72.4)	62 (27.6)	25.827	<0.001
	Poor	1078 (74.5)	369 (25.5)		
	Average	1807 (80.7)	432 (19.3)		
	Rich	43 (86.0)	7 (14.0)		
The way parents educate their children	Strict control type	232 (68.6)	106 (31.4)	50.717	<0.001
	Hands-off type	136 (65.7)	71 (34.3)		
	Democratic deliberative type	2682 (80.0)	670 (20.0)		
	Doting and pampering type	41 (64.1)	23 (35.9)		
Father’s academic credentials	High school degree and below	2470 (77.2)	728 (22.8)	6.238	0.044
	Bachelor’s degree	576 (81.5)	131 (18.5)		
	Master’s degree and above	45 (80.4)	11 (19.6)		
Mother’s academic credentials	High school degree and below	2624 (77.7)	754 (22.3)	1.736	0.42
	Bachelor’s degree	445 (80.0)	111 (20.0)		
	Master’s degree and above	22 (81.5)	5 (18.5)		
The relationship between parents	Marginalized and alienated	40 (56.3)	31 (43.7)	78.727	<0.001
	Flat and common	554 (69.8)	240 (30.2)		
	Harmonious	1588 (78.5)	434 (21.5)		
	Very affectionate	909 (84.6)	165 (15.4)		
University requires students to report daily information	No	228 (71.9)	89 (28.1)	7.509	0.006
	Yes	2863 (78.6)	781 (21.4)		
University carries out popular science propaganda	No	230 (69.1)	103 (30.9)	17.055	<0.001
	Yes	2861 (78.9)	767 (21.1)		
University conducts distance learning	No	461 (74.4)	159 (25.6)	5.811	0.016
	Yes	2630 (78.7)	711 (21.3)		
University provides psychological counseling	No	952 (73.5)	344 (26.5)	23.564	<0.001
	Yes	2139 (80.3)	526 (19.7)		

**Table 4:** Four logistic regression analysis to assess the correlation between selected characteristics and PTSD occurrence.

Variables	RM1			RM2			RM3			RM4		
	OR	95% CI	*p*	OR	95% CI	*p*	OR	95% CI	*p*	OR	95% CI	*p*
Gender												
Male	ref	ref	ref	ref	ref	ref	ref	ref	ref	ref	ref	ref
Female	0.601	0.513–0.704	<0.001	0.608	0.518–0.712	<0.001	0.628	0.534–0.738	<0.001	0.621	0.528–0.731	<0.001
Major												
Engineering	ref	ref	ref	ref	ref	ref	ref	ref	ref	ref	ref	ref
Science	1.244	0.986–1.568	0.065	1.221	0.967–1.541	0.093	1.275	1.006–1.615	0.044	1.253	0.986–1.592	0.065
Agriculture	1.662	1.117–2.473	0.012	1.639	1.099–2.444	0.015	1.656	1.100–2.492	0.016	1.693	1.123–2.552	0.012
Liberal art	1.392	1.104–1.756	0.005	1.39	1.101–1.754	0.006	1.476	1.165–1.871	0.001	1.499	1.182–1.902	0.001
Medicine	1.571	1.026–2.407	0.038	1.609	1.050–2.467	0.029	1.579	1.020–2.446	0.041	1.614	1.041–2.501	0.032
Art	1.669	1.278–2.180	<0.001	1.648	1.261–2.155	<0.001	1.744	1.327–2.292	<0.001	1.76	1.337–2.316	<0.001
Student leader												
No	ref	ref	ref	ref	ref	ref	ref	ref	ref	ref	ref	ref
Yes	0.748	0.624–0.896	0.002	0.761	0.635–0.912	0.003	0.809	0.673–0.973	0.025	0.835	0.693–1.005	0.057
Confirmed cases in residential villages/communities												
No				ref	ref	ref	ref	ref	ref	ref	ref	ref
Yes				1.346	1.024–1.770	0.033	1.299	0.983–1.716	0.066	1.291	0.976–1.708	0.074
Imported cases from abroad in residential village/community												
No				ref	ref	ref	ref	ref	ref	ref	ref	ref
Yes				1.86	1.242–2.784	0.003	1.765	1.171–2.660	0.007	1.725	1.143–2.602	0.009
Imported cases from abroad in residential township/town/street												
No				ref	ref	ref	ref	ref	ref	ref	ref	ref
Yes				0.978	0.734–1.303	0.879	0.996	0.745–1.331	0.976	0.988	0.739–1.321	0.936
Family type												
Nuclear family							ref	ref	ref	ref	ref	ref
Extended family							0.974	0.695–1.367	0.88	0.977	0.696–1.372	0.894
Single-parent families							1.441	0.849–2.447	0.176	1.47	0.866–2.496	0.154
Remarried family							1.613	0.567–4.592	0.371	1.625	0.569–4.640	0.364
Family’s financial situation												
Extremely poor							ref	ref	ref	ref	ref	ref
Poor							1.048	0.751–1.461	0.784	1.031	0.739–1.439	0.857
Average							0.814	0.583–1.138	0.229	0.818	0.585–1.144	0.241
Rich							0.673	0.281–1.614	0.375	0.667	0.278–1.603	0.366
The way parents educate their children												
Arbitrary control type							ref	ref	ref	ref	ref	ref
Uninvolved and permissive type							1.028	0.702–1.506	0.885	1.031	0.703–1.511	0.877
Democratic deliberative type							0.714	0.552–0.924	0.011	0.73	0.564–0.946	0.017
Doting and pampering type							1.507	0.847–2.682	0.163	1.487	0.834–2.648	0.178
Father’s academic credentials												
Senior high school degree and below							ref	ref	ref	ref	ref	ref
Bachelor’s degree							0.894	0.715–1.116	0.322	0.905	0.724–1.131	0.379
Master’s degree and above							0.922	0.461–1.846	0.819	0.934	0.466–1.870	0.847
The relationship between parents												
Marginalization and alienation							ref	ref	ref	ref	ref	ref
Flat and common							0.607	0.363–1.015	0.057	0.589	0.351–0.986	0.044
Harmony							0.439	0.264–0.732	0.002	0.427	0.256–0.713	0.001
Very affectionate							0.313	0.184–0.531	<0.001	0.309	0.182–0.526	<0.001
University requires students to report daily information												
No										ref	ref	ref
Yes										0.842	0.639–1.110	0.223
University carried out popular science propaganda												
No										ref	ref	ref
Yes										0.784	0.595–1.034	0.084
University conducts distance learning												
No										ref	ref	ref
Yes										1.004	0.800–1.260	0.97
University provides psychological counseling												
No										ref	ref	ref
Yes										0.766	0.638–0.918	0.004

## Data Availability

Data are available upon reasonable request. zyy15809057928@outlook.com.
